# Reprioritization of biofilm metabolism is associated with nutrient adaptation and long-term survival of *Haemophilus influenzae*

**DOI:** 10.1038/s41522-019-0105-6

**Published:** 2019-11-05

**Authors:** Alistair Harrison, Rachael L. Hardison, Rachel M. Wallace, James Fitch, Derek R. Heimlich, Meghan O’ Bryan, Laura Dubois, Lisa St. John-Williams, Robert P. Sebra, Peter White, M. Arthur Moseley, J. Will Thompson, Sheryl S. Justice, Kevin M. Mason

**Affiliations:** 1 The Abigail Wexner Research Institute at Nationwide Children’s Hospital, Center for Microbial Pathogenesis, 700 Children’s Drive, Columbus, OH 43205 USA; 2 The Abigail Wexner Research Institute at Nationwide Children’s Hospital, The Steve and Cindy Rasmussen Institute for Genomic Medicine, 575 Children’s Crossroad, Columbus, OH 43215 USA; 3 Duke Proteomics and Metabolomics Core Facility, Duke Center for Genomic and Computational Biology, Duke University, 701 West Main Street, Durham, NC 27701 USA; 4 Icahn School of Medicine at Mount Sinai, Icahn Institute and Department of Genetics & Genomic Sciences, 1 Gustave L. Levy Place, New York, NY 10029 USA; 5 Infectious Diseases Institute, The Ohio State University College of Medicine, 700 Children’s Drive, Columbus, OH 43205 USA

**Keywords:** Bacteriology, Bacteriology, Biofilms, Biofilms

## Abstract

Nontypeable *Haemophilus influenzae* (NTHI) is a human-restricted pathogen with an essential requirement for heme–iron acquisition. We previously demonstrated that microevolution of NTHI promotes stationary phase survival in response to transient heme–iron restriction. In this study, we examine the metabolic contributions to biofilm formation using this evolved NTHI strain, RM33. Quantitative analyses identified 29 proteins, 55 transcripts, and 31 metabolites that significantly changed within in vitro biofilms formed by RM33. The synthesis of all enzymes within the tryptophan and glycogen pathways was significantly increased in biofilms formed by RM33 compared with the parental strain. In addition, increases were observed in metabolite transport, adhesin production, and DNA metabolism. Furthermore, we observed pyruvate as a pivotal point in the metabolic pathways associated with changes in cAMP phosphodiesterase activity during biofilm formation. Taken together, changes in central metabolism combined with increased stores of nutrients may serve to counterbalance nutrient sequestration.

## Introduction

In the wake of the success of the *Haemophilus influenzae* type b (Hib) vaccine, infections caused by nontypeable *H. influenzae* (NTHI) strains have increased dramatically, and now represent a major cause of otitis media (OM), exacerbations of chronic obstructive pulmonary disease, and bacterial sinusitis. Although an innocuous commensal of the nasopharynx, under nutrient stress, viral infection, or other environmental factors, NTHI can become an invasive pathogen. Migration of NTHI from the nasopharynx to the upper and lower respiratory tract coincides with initiation of multiple diseases.^1–9^ The burden of OM is substantial. Children receive three times more antibiotics than adolescents/adults, with 40% of all antibiotics prescribed for the treatment of OM.^10,11^ As a result, the emergence of antibiotic-resistant strains and the cost associated with managing OM exceeds $5 billion annually in the U.S.^12–15^ Worldwide, OM is also one of the most common indications for outpatient surgery in children,^16^ and the most common cause of hearing loss leading to developmental delays in behavior, language, and education.^17–20^

The pathogenesis-related factors that lead to hearing loss and other sequelae as a consequence of OM are, however, understudied. In addition, bacterial and host elements that dictate the complexity and fitness of NTHI, the impact on NTHI persistence and recurrence, and how chronic sequelae arise from OM are not completely understood. Clinical management of this highly prevalent disease has therefore relied heavily on antibiotic therapies.^21–23^ Thus, the delineation of host-pathogen mechanisms that lead to OM-derived sequelae will inform the development of new therapeutic approaches.

Host microenvironmental influences on NTHI pathoadaptation and evolution have revealed additional targets for treatment of OM, based upon the multiple pathogenic lifestyles revealed in our studies (e.g., biofilm, intracellular).^24^ In animal models of experimental OM and in clinical samples, formation of an organized biofilm is an observed NTHI pathogenic lifestyle.^25–28^ The development of structured biofilms in other pathogens is conditional upon availability of essential nutrients.^29–31^ In our previous studies, we observed striking changes in the architecture and morphology of NTHI biofilms due to transient heme–iron restriction. Specifically, transiently heme-restricted NTHI biofilms formed tall towers composed of a filamentous morphology, while biofilms continuously exposed to heme–iron displayed a mat-like architecture with distinct coccoid morphology.^32^ To determine potential roles of transient heme–iron restriction on NTHI survival, we established long-term parallel cultures that were continuously exposed to heme–iron or transiently restricted of heme–iron for 24 h. In these studies, we observed that transient heme–iron restriction promotes extended stationary phase survival of NTHI in vitro, as compared with NTHI continuously exposed to heme–iron.^33^ We isolated an NTHI strain adapted to long-term survival following transient heme–iron depletion on day 33, termed RM33. Whole-genome sequencing revealed a sole single nucleotide polymorphism, found in *icc*, a gene that encodes the cAMP phosphodiesterase in RM33. Structural modeling and biochemical studies indicated that mutant proteins of Icc failed to hydrolyze cAMP.^33^ The RM33 strain displayed phenotypes associated with subclinical OM with an increase in intracellular bacterial community development early during experimental OM after clinical resolution of disease.^33^

Multiple gaps remain in our knowledge of how nutritional sequestration dictates microbial virulence factors that equip survival of bacteria in the host. We propose that the microevolution that occurs in response to transient nutrient limitation is associated with physiological differences in RM33 biofilms, a known lifestyle during OM pathogenesis. Thus, we have taken a systems biology approach to delineate the physiological and metabolic profiles of RM33 during biofilm growth. To identify bacterial factors that may be contributing to the long-term survival and distinct phenotypes of transiently restricted NTHI, we investigated the proteome, transcriptome, and metabolome of biofilms formed by RM33 compared with those formed by the parental strain. These data reveal an increase in proteins and metabolites related to many of the central metabolic processes in RM33, suggesting that metabolism plays a key role in the ability of NTHI to survive in both long-term cultures and in the host.^33^ The metabolic changes observed are consistent with those observed in other persistent microorganisms and in intracellular survival. In-depth analysis of the physiological and metabolic status of a persistent isolate of NTHI in the biofilm lifestyle provides a platform to determine previously undescribed pathogenic mechanisms that contribute to NTHI persistence during OM and other biofilm-mediated diseases. Identification of new therapeutic strategies requires a more detailed molecular description of the NTHI physiology associated with persistence during disease.

## Results

### Transient heme–iron restriction promotes microevolution leading to increased biofilm height

**Fig. 1:**
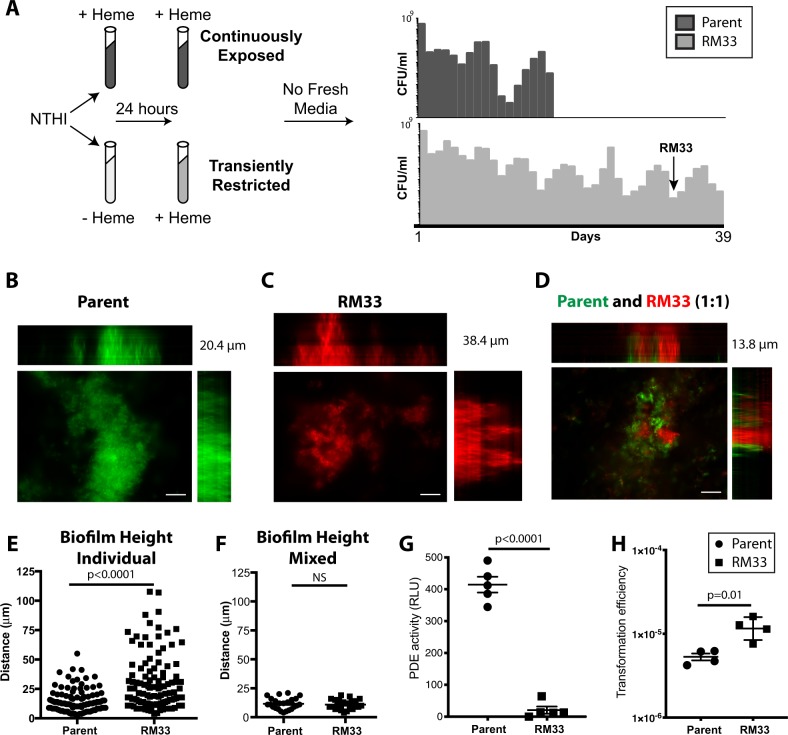
A persistent isolate of NTHI exhibits increased biofilm height and transformation efficiency. **a** Schematic representation of environmental heme–iron restriction. 86-028NP was cultured in defined iron source (DIS) medium in the presence (+) or absence (−) of 2 µg/mL heme–iron for 24 h to generate “replete” or “restricted” populations, respectively, adapted from Hardison et al.^33^ These two populations were subcultured into DIS medium containing 2 µg/mL heme–iron. The “continuously exposed” or “transiently restricted” cultures were continuously incubated under static microaeration with no fresh medium added. Viability was determined by plating for CFU/mL every 24 h, each bar indicates the CFU on that day for a representative experiment. The persistent isolate, RM33, was isolated from the transiently restricted culture on day 33 (arrow). **b** Representative image of a biofilm of 86-028NP (pGM1.1) grown for 48 h with orthogonal views of the three-dimensional rendering of a 20.4 µm high biofilm. **c** Representative image of a biofilm of RM33 (pKM1.1) grown for 48 h with orthogonal views of the three-dimensional rendering of a 38.4 µm high biofilm. **d** Representative image of a 13.8 µm biofilm with a 1:1 mixture of 86-028 NP (pGM1.1) and RM33 (pKM1.1) grown for 48 h with orthogonal views of the three-dimensional rendering. **e** Quantitative assessment of the height of biofilms formed by either the parental strain [86-028NP (pGM1.1), circle] and [RM33 (pKM1.1), square]. The height of the biofilm was measured at ten random locations in technical duplicate and performed on six independent occasions (*n* = 120 per strain). Each point represents an individual measurement. Statistical significance was determined by a two-tailed unpaired Student’s *t* test. **f** Quantitative assessment of the height of each strain within a mixed biofilm was measured as described in **e**. **g** The phosphodiesterase activity of whole-cell lysates prepared from 48 h biofilms was determined as described in “Methods” section. The mean and standard error of the mean (s.e.m.) are indicated for the results of five independent assays. Statistical significance was determined using a two-tailed unpaired Student’s *t* test. **h** Exogenous DNA was added to a 48 h biofilm of either 86-028NP or RM33. Biofilms were physically disrupted and the efficiency of DNA uptake is reported as the number of antibiotic-resistant colonies within the total population. The mean and standard error of the mean (s.e.m.) are indicated for the results of four independent assays. Statistical significance was determined using a two-tailed unpaired Student’s *t* test. Scale bar indicates 25 μm

We previously demonstrated that transient depletion of the essential micronutrient heme–iron increases the longevity of NTHI survival in vitro as compared with cultures that were continuously exposed to heme–iron (Fig. 1a; ^33^). Whole-genome sequencing of an isolate obtained on day 33 (termed RM33) of a long-standing stationary phase culture revealed a single mutation within the *icc* gene that encodes the only known cAMP phosphodiesterase in NTHI strain 86-028NP.^33^ Given the observation that biofilms are an important pathogenic lifestyle of NTHI during OM,^28,34,35^ we investigated the role of *icc* in biofilm formation. The parental strain formed biofilms with a mat-like architecture with a range in overall height of 2.5–55.1 µm (mean = 14.95 ± 9.678) (Fig. 1b, e). In marked contrast, the RM33 mutant strain formed biofilms with significantly increased height of 3.8–107.7 µm (mean = 30.82 ± 23.6; *p* < 0.0001), hallmarked by the presence of towers (Fig. 1c, e). Interestingly, coculture of RM33 with the parental strain resulted in segregation of the strains within the biofilms with a reduction in total biofilm height (Fig. 1d, f), suggesting an influence of the presence of the parental strain on RM33 architecture.

The *icc* mutation in RM33 disrupts one of two iron cofactors within the active site and significantly reduces cAMP phosphodiesterase activity of planktonic bacteria.^33^ To determine the amount of cAMP phosphodiesterase activity during biofilm formation, whole-cell lysates of parental and RM33 biofilms were analyzed. The RM33 biofilms displayed a nearly complete abrogation of phosphodiesterase activity (Fig. 1g). Although the exact mechanisms remain unclear, exogenous cAMP significantly increases the efficiency of DNA uptake by *H. influenzae* under conditions of nutrient depravation.^36^ Therefore, we hypothesize that the ability of the parent to reduce exogenously available cAMP through import and degradation would also decrease intracellular cAMP within RM33. Taken together, our data suggest that cAMP serves as a soluble second messenger that regulates biofilm architecture.

### Biofilms formed by RM33 exhibit significant differences in transcription and protein levels of the competence regulon

Due to the phenotypic changes in biofilm formation of the evolved strain RM33, we took a systems biology approach to examine global changes associated with decreased phosphodiesterase activity. To identify bacterial factors that may contribute to the distinct phenotypes of RM33,^33^ we compared proteomic, transcriptomic, and metabolomic profiles of biofilms formed by RM33 and the parental strain using high-resolution quantitative two-dimensional liquid chromatography-tandem mass spectrometry (2D LC-MS/MS) and RNASeq. We observed statistically significant differences in 29 proteins, 55 transcripts, and 31 metabolites in biofilms formed by RM33 (Table 1). Interestingly, 68% of the upregulated proteins have known roles in competence. Consistent with the observed increase in proteins and transcripts associated with competence, the transformation efficiency of RM33 biofilms were significantly increased as compared with the parent (Fig. 1h).

**Table 1:** Significant changes in protein and RNA transcripts detected in RM33 and the parental strain biofilms

NTHI gene #/Name		Function	Protein (fold change)	*p* value	RNASeq - mean FC	*p* value
NTHI1768	TrpE	Anthranilate synthase component I	9.1	0.0000003	1.8	1.2E−06
NTHI1763	TrpC	Bifunctional indole-3-glycerol phosphate synthase/phosphoribosylanthranilate isomerase	6.9	0.00012	1.5	1.3E−06
NTHI1702	TrpB	Tryptophan synthase subunit beta	6.9	0.00004	0.4	7.8E−03
NTHI1810	MalQ	Alpha-glucanotransferase	5.3	0.00001	0.3	7.5E−02
NTHI1701	TrpA	Tryptophan synthase subunit alpha	4.8	0.0002	0.1	1.3E−01
NTHI1920	Mao2	Malic enzyme	4.3	0.000003	0.2	8.1E–03
NTHI0202	HemR	Hemin receptor	4.3	0.0004	NS	NS
NTHI0623	AphA	Acid phosphatase/phosphotransferase	4.0	0.000003	NS	NS
NTHI0632	RbsB	D-ribose transporter subunit RbsB	3.8	0.000002	1.6	2.9E−04
NTHI0232		Sialic acid transporter, TRAP-type C4-dicarboxylate transport system, periplasmic component	3.6	0.00003	0.0	9.4E−01
NTHI1707		ABC transporter periplasmic protein	3.5	0.00001	NS	NS
NTHI0398	SdaC	Serine transporter	3.1	0.00002	1.8	9.3E−03
NTHI1964	SucA	Oxoglutarate dehydrogenase E1	3.0	0.00001	1.8	2.7E−04
NTHI0397	SdaA	l-serine dehydratase	3.0	0.000005	2.1	2.9E−04
NTHI1809	GlgB	Glycogen branching enzyme	3.0	0.00005	2.0	4.2E−03
NTHI0235		Acetylneuraminic acid mutarotase	2.8	0.00003	2.0	7.7E−05
NTHI1764	TrpD	Anthranilate phosphoribosyltransferase	2.8	0.0001	0.3	1.8E−09
NTHI1295		Carbon starvation protein, membrane protein	2.7	0.0001	1.9	2.7E−04
NTHI1806	GlgA	Glycogen synthase	2.5	0.0002	2.0	2.1E−04
NTHI0647		Hypothetical protein	2.4	0.00005	NS	NS
NTHI0660		Aspartate ammonia-lyase	2.3	0.00004	0.0	1.8E−01
NTHI1808	GlgX	Glycogen operon protein	2.2	0.00004	2.1	3.7E−04
NTHI1816	Cdd	Cytidine deaminase	2.2	0.0004	0.0	5.2E−01
NTHI1903		Folylpolyglutamate synthase	2.1	0.0006	NS	NS
NTHI0610	AtpG	ATP synthase F0F1 subunit gamma	2.1	0.0002	NS	NS
NTHI0973	PckA	Phosphoenolpyruvate carboxykinase	2.0	0.0002	0.1	2.7E−01
NTHI0088	NrdD	Anaerobic ribonucleoside triphosphate reductase	2.0	0.0002	NS	NS
NTHI1028	ClpB	ClpB chaperone	−2.1	0.00003	NS	NS
NTHI0306	AroB	3-dehydroquinate synthase	−3.4	0.00006	NS	NS

### Amino acids and enzymes associated with central metabolism are significantly increased in biofilms produced by RM33

**Fig. 2:**
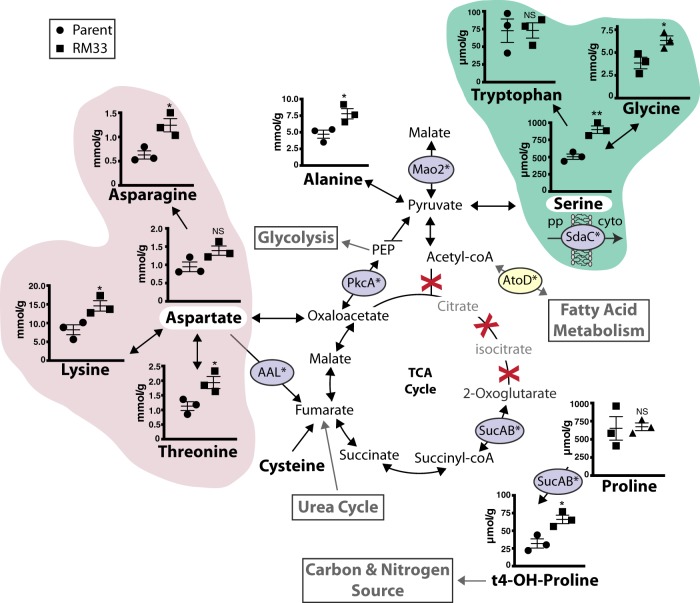
Amino acid metabolism and entry into the TCA cycle during biofilm growth is increased in RM33 biofilms. The pathways of amino acids that are associated with the TCA cycle were determined from the information provided for 86-028NP in the Kyoto Encyclopedia of Genes and Genomes (KEGG) and Biocyc Pathway databases. The concentration of amino acids within the biofilms of 86-028NP (circle) and RM33 (square) were quantitated as described in “Methods” section. The red X indicates absence of known orthologs of enzymes involved in the TCA cycle for 86-028NP. The enzymes encircled in purple are significantly increased in RM33 biofilms. The enzyme encircled in yellow is significantly decreased in RM33 biofilms. The concentrations of aspartate (pink cloud) and serine (green cloud) in the spent media were below the level of detection (indicated by the white highlight). pp periplasm, cyto cytoplasm. The mean and s.e.m. are depicted for each metabolite from three independent biological biofilms grown for 48 h. Statistical significance was determined using a two-tailed unpaired Student’s *t* test (*p < 0.036; **p = 0.004)

Amino acids were quantified from both the spent media and bacteria of biofilms grown on abiotic surfaces for 48 h. In most cases, there was no significant difference in the concentration of each amino acid between the spent and fresh media (Supplementary Fig. 3). For serine and aspartate, these amino acids were below the limit of detection, despite replenishing the medium after 24 h (Supplementary Fig. 3). Interestingly, serine can be converted to pyruvate for biosynthesis of alanine (significantly increased in RM33 biofilms) or for entry into fatty acid metabolism via acetyl-coA. However, the enzyme AtoD is significantly decreased in RM33 biofilms, suggesting that the preferred utilization of serine is for conversion to alanine or malate as an entry point into the tricarboxylic acid (TCA) cycle (Fig. 2). Aspartate can directly be shuttled into the TCA cycle through conversion to oxaloacetate (Fig. 2). Serine and aspartate are key intermediates in the biosynthesis of other amino acids, most of which are significantly increased in RM33 (Fig. 2 pink and green clouds). Cysteine can also be shuttled into the TCA cycle via fumarate, but was not evaluated in this study. As with other *H. influenzae* strains, 86-028NP does not contain known orthologs for the enzymes required for the entry of acetyl-coA into the TCA cycle (missing enzymes: citrate synthase, aconitate hydratase, isocitrate dehydrogenase) (Fig. 2). Therefore, the depletion of serine and aspartate suggests important roles for these amino acids as entry points into central metabolism of NTHI during biofilm formation through pyruvate and oxaloacetate, respectively.

Significant differences were observed in the amounts of amino acids that are synthesized through serine and aspartate intermediates in the biofilms formed by RM33 (i.e., asparagine, lysine, threonine via aspartate; glycine, tryptophan via serine) (Fig. 2). From our concurrent protein analysis, we observed increased production of the SdaC serine transporter (see Table 1) in RM33 biofilms, suggesting that the observed increase in biofilm-associated serine in RM33 may be, in part, due to increased import into the bacterium. In contrast, accumulation of aspartate does not appear to be mediated by import, since we did not observe increased production of the glutamate/aspartate ABC transporter.

The proteomic analysis revealed three enzymes that are significantly increased in RM33 biofilms that produce metabolites as additional entry points into the TCA cycle. The conversion of aspartate to fumarate is catalyzed by aspartate ammonia-lyase (AAL) (Fig. 2). Production of the malic enzyme (Mao2) that converts pyruvate to malate (TCA cycle) was significantly increased (4.3-fold) in the biofilms of RM33 (Fig. 2). Phosphoenolpyruvate carboxykinase (PckA) is an important enzyme in the coordination of central metabolism that intersects glycolysis/gluconeogenesis with the TCA cycle and fumarate metabolism and was also significantly increased (twofold) in RM33 biofilms (Fig. 2).

The protein and transcriptional analyses reveal additional metabolic differences between RM33 and the parental strain. In addition to the production of succinate, the 2-oxoglutarate dehydrogenase enzyme, SucAB, converts proline, and 2-oxoglutarate to trans-4-hydroxyproline (Fig. 2). Both the SucAB enzyme and the trans-4-hydroxyproline product were significantly increased in the biofilms of RM33 as compared with the parent (Fig. 2). Although trans-4-hydroxyproline can also be produced by non-enzymatic hydroxylation of proline by reactive oxygen species,^37^ the increase in SucAB observed suggests trans-4-hydroxyproline is produced enzymatically. Hydroxyproline is a source of carbon and nitrogen. The transcription of *atoD* was significantly reduced (2.5-fold) in the biofilms formed by RM33. This enzyme catalyzes an early step in fatty acid metabolism by releasing the lipid moiety from acetyl-coA. A reduction in the amount of *atoD* transcription therefore suggests that fatty acid metabolism is decreased in RM33.

### Metabolites associated with the urea cycle are significantly increased in biofilms formed by RM33

**Fig. 3:**
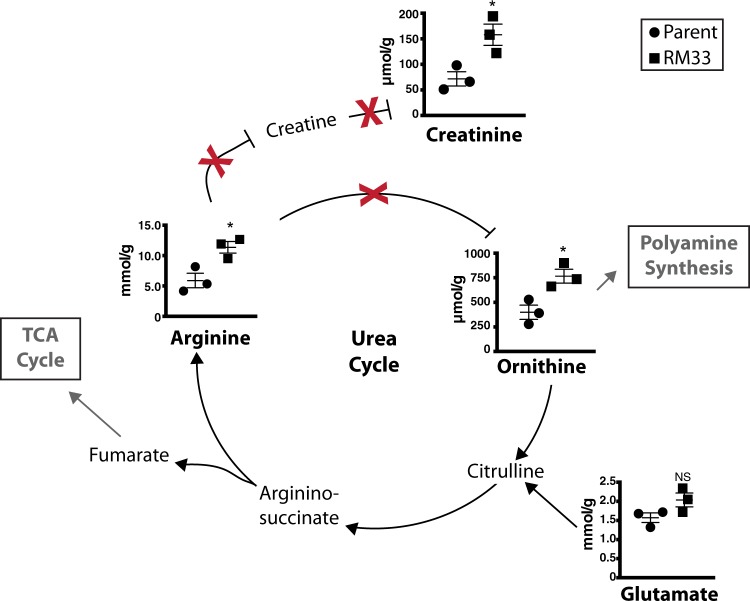
Metabolites associated with the urea cycle are significantly increased in RM33 biofilms. The concentrations of the metabolites associated with the urea cycle determined from information at KEGG and Biocyc were quantified as described in “Methods” section. The red X indicates absence of known orthologs of enzymes involved in the urea cycle for 86-028NP. The mean and s.e.m. of the concentrations for the metabolites from three independent biological replicates of 86-028NP (circle) and RM33 (square) 48 h biofilms are presented. Statistical significance was determined using a two-tailed unpaired Student’s *t* test (*p < 0.025)

As discussed above, fumarate is an important entry point into the TCA cycle from amino acid metabolism (e.g., aspartate and cysteine). Fumarate is also a byproduct of the urea cycle, generated during the conversion of L-arginosuccinate to arginine. Although we also observe differences in ornithine, there is no known ortholog of arginase in the strains used here. Therefore, significant increases in arginine and ornithine suggest there are alterations in the urea cycle that modulate nitrogen metabolism differently between 86-028NP and RM33 (Fig. 3).

Biogenic amines play multiple roles in bacterial physiology and pathogenesis.^38^ As with the amino acids, there were no significant differences in the concentrations of biogenic amines in the spent biofilm supernatants from 86-028NP and RM33 (Supplementary Fig. 4). However, the RM33 biofilms contained higher levels of creatinine (Fig. 3). Creatinine is a biogenic amine formed from the metabolism of arginine (also increased in RM33) as part of the urea cycle (Fig. 3). The biological role of creatinine in bacterial physiology is not understood.

### Polyamine biosynthesis is differentially regulated in biofilms formed by the parent and by RM33

**Fig. 4:**
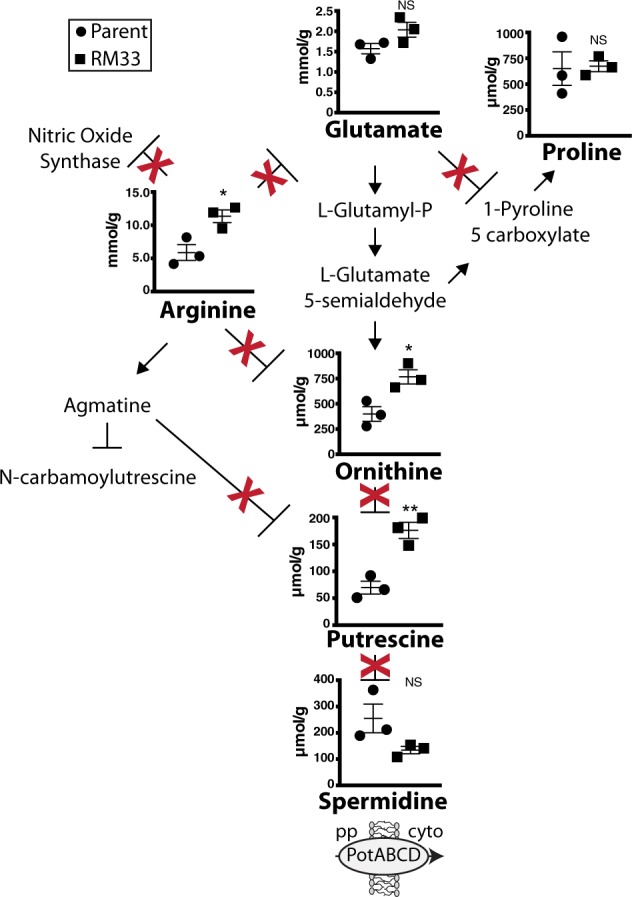
Production of polyamines differs between 86-028NP and RM33 biofilms. The classical pathways for polyamine synthesis were adapted from KEGG. The red X indicates absence of known orthologs of enzymes involved in polyamine synthesis for 86-028NP. The mean and s.e.m. of the concentrations of the metabolites within three independent biological replicates 86-028NP (circle) and RM33 (square) 48 h biofilms were quantified as described in “Methods” section. Statistical significance was determined using a two-tailed unpaired Student’s *t* test (*p < 0.02; **p = 0.005). pp periplasm, cyto cytoplasm

Ornithine from the urea cycle is also a key entry point into polyamine synthesis. Polyamines are small polycationic molecules that have recently been investigated for roles in modulation of bacterial virulence.^38^ Glutamate appears to be a metabolite that is used to produce ornithine (Fig. 4). Although the enzymes responsible for polyamine biosynthesis in NTHI are unknown (Supplementary Table 3), we observed significant increases in putrescine within RM33 biofilms. Although not significant, we observe a trend for an increase in spermidine within the biofilms formed by the parent (Fig. 4).

### Proteomic analysis reveals increased tryptophan biosynthetic pathway in biofilms formed by RM33 biofilms

**Fig. 5:**
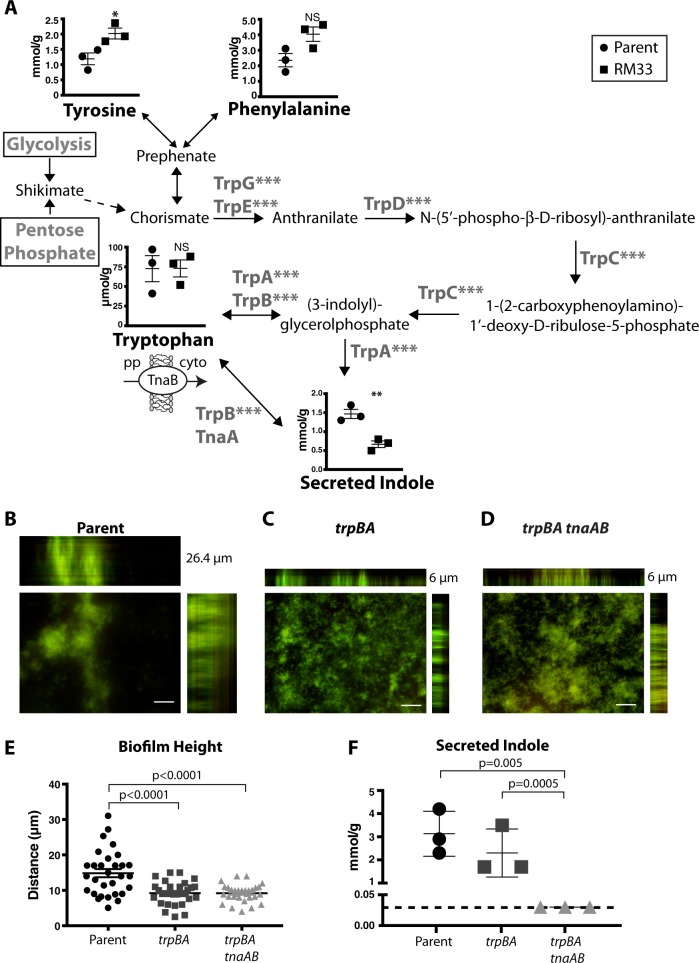
Tryptophan biosynthesis enzymes are significantly increased in RM33 biofilms. **a** The enzymatic pathway for synthesis of tryptophan was adapted from KEGG. The concentration of amino acids within the biofilms of 86-028NP (circle) and RM33 (square) and secreted indole were quantitated from three independent biological replicates as described in the “Methods” section. The TnaB importer of tryptophan from the periplasm (pp) to the cytoplasm (cyto) was not significantly different between the two strains. Statistical significance was determined using a two-tailed unpaired Student’s *t* test (*p = 0.03; **p = 0.007; ***p < 0.0001). **b** Representative image of a biofilm of the parent (86-028NP) grown for 48 h and stained with LIVE/DEAD to visualize DNA stain with orthogonal views of the three-dimensional rendering of a 26.4 µm biofilm. **c** Representative image of a biofilm of 86-028NP *trpBA* mutant grown for 48 h and stained with LIVE/DEAD to visualize bacteria with orthogonal views of the three-dimensional rendering of a 6 µm biofilm. **d** Representative image of a biofilm of 86-028NP *trpBA tnaAB* mutant grown for 48 h and stained with LIVE/DEAD to visualize bacteria with orthogonal views of the three-dimensional rendering of a 6 µm biofilm. **e** Quantitative assessment of the height of biofilms formed by the parental strain (86-028 NP), 86-028NP *trpBA*, or 86-028NP *trpBA tnaAB*. The height of the biofilm was measured at ten random locations on three independent occasions (*n* = 30 per strain). Each point represents an individual measurement. Statistical significance was determined by a two-tailed unpaired Student’s *t* test. **f** The culture supernatant from three biofilms grown for 48 h on three independent occasions were used to quantify the level of indole as described in the “Methods” section. The dashed horizontal line indicates the limit of detection. The mean and s.e.m. are reported. Statistical significance was determined using a two-tailed unpaired Student’s *t* test. Scale bar indicates 25 μm

All of the proteins involved in tryptophan biosynthesis (TrpABCDEG) were increased in biofilms formed by RM33 (Fig. 5a). Phenylalanine and tyrosine are substrates for synthesis of tryptophan via chorismate (Fig. 5a). Once synthesized, or imported through the tryptophan transporter, TnaB, tryptophan can be catabolized by tryptophanase, encoded by *tnaA*, to produce indole. Indole is an aromatic metabolite with diverse functions and roles in signaling, biofilm formation, and persistence.^39^ Interestingly, secreted indole was significantly decreased in biofilms formed by RM33 (Fig. 5a).

To test the contribution of tryptophan biosynthesis to the architectural changes observed in RM33, the genes encoding the terminal enzymes in tryptophan and indole biosynthesis (*trpBA tnaA*) as well as the ability to import tryptophan (*tnaB*) were deleted, resulting in a tryptophan auxotroph. Despite the similarity in growth rates and viability of the mutant strains (Supplementary Fig. 6), deletion of *trpBA* (no tryptophan synthesis) or *trpBAtnaAB* (no tryptophan/indole synthesis, impaired tryptophan import) led to a significant reduction in the overall biofilm height (Fig. 5b–e). However, a significant reduction in secreted indole was only observed in the *trpBAtnaAB* quadruple mutant (Fig. 5f). These data suggest that tryptophan plays an essential role during biofilm formation. The transcription of the tryptophan transporter, *mtr*, was significantly increased in RM33 biofilms (Table 1), suggesting that Mtr may transport tryptophan in the absence of TnaB to be metabolized into other bioactive molecules. However, our knowledge of the specific role of tryptophan synthesis and indole export in NTHI pathogenesis is limited.

## Discussion

Nutritional sequestration promotes evolutionary changes for pathoadaptation that impact multiple bacterial lifestyles during disease. NTHI resides as a commensal in the nasopharynx but under permissive conditions can migrate into the middle ear.^40,41^ The host tightly sequesters heme–iron and other essential nutrients. Over the course of infection, inflammation and immune responses cause fluctuations in iron availability. NTHI upregulates core iron- and heme–responsive genes during experimental OM,^42^ suggesting a heme–iron limited environment. Moreover, NTHI strains isolated from the middle ears of children with OM contain more heme acquisition genes than strains isolated from the throats of healthy children,^43^ further suggesting the important role for heme–iron acquisition during disease.

A common pathogenic lifestyle of many bacteria is the formation of organized biofilm communities. Here, we provide evidence that adaptation to nutritional limitation through evolution alters biofilm architecture and long-term survival of NTHI at least, in part, through changes in global metabolism. In this study, we have taken a systems biology approach to elucidate the unique proteomic, transcriptomic and metabolic profiles of the RM33 persistent NTHI isolate (obtained during long-stationary phase survival in response to iron limitation) in the biofilm lifestyle. We have demonstrated an important role for tryptophan biosynthesis in development of the overall biofilm architecture. In addition, we observed an overall shift in the metabolic state of a persistent isolate, RM33, toward more efficient biosynthesis of amino acids and DNA uptake, with pyruvate being an important central metabolite in the orchestration of metabolic processes. Moreover, the polyamine and indole profiles of RM33 and the parent are unique, providing additional insight into the potential mechanisms of long-term survival of RM33.

We observed that production of all enzymes for tryptophan biosynthesis are significantly increased in RM33 biofilms. Mutations in the terminal step of tryptophan biosynthesis dramatically reduces the overall height and architecture of NTHI biofilms (see Fig. 5). There is precedent for the role of tryptophan during pathogenesis in multiple organisms. Modulation of tryptophan biosynthesis commonly occurs during biofilm formation by multiple pathogens.^44^ *Chlamydia* also responds to iron starvation through changes in metabolism, which further promotes intracellular survival. These responses include increased expression of genes with roles in tryptophan biosynthesis, possibly as a protective measure against the degradation of tryptophan by dendritic cell-produced indoleamine 2, 3-dioxygenase. Similar alterations in tryptophan metabolism also occur in *Francisella* and uropathogenic *Escherichia coli*.^45–50^ In NTHI, there is a coordination between the production of the ToxA/VapA toxin/antitoxin system and tryptophan biosynthesis, suggesting that responses to stress through ToxA/VapA is multifactorial and metabolism mediated.^51^ Taken together with our prior studies,^33^ tryptophan biosynthesis appears to contribute to both biofilm formation and IBC development, indicators of persistence of NTHI during OM.

For obligate intracellular pathogens, the ability to store amino acids, particularly tryptophan, is considered a mechanism to persist in the intracellular environment.^45,46^ Our published studies indicate that RM33 establishes intracellular bacterial communities at time points earlier than the parental strain,^33^ which is consistent with a role of tryptophan biosynthesis in intracellular persistence. Taken together, we hypothesize that tryptophan biosynthesis appears to be important for both luminal biofilm formation and intracellular bacterial community development.

Our observation that despite increased production of enzymes involved in tryptophan biosynthesis, RM33 biofilms produce significantly less indole than the parental biofilms is intriguing as the relationship between cAMP levels and indole signaling has been linked to bacterial persistence. Persistence in *E. coli* is linked to a reduction in indole which occurs via loss of tryptophanase activity through either a cAMP phosphodiesterase or a toxin/antitoxin system.^52^ However, when tryptophanase activity is abolished, persistence of *E. coli* is decreased,^53^ suggesting that the concentration of indole is important in modulating persistence. Indole can be an important regulator of biofilm formation, although the effect of biofilm formation may be dependent on whether or not the bacterium produces indole endogenously.^54^ Endogenous oxidative stress increases tryptophanase activity, increases indole and delays biofilm formation.^55^ Consistent with our observations, decreases in indole coincides with increased biofilm formation.^52^ Ongoing studies will investigate the role of changes in indole concentrations on persistence of NTHI during experimental OM.

The increase in competence exhibited by RM33 biofilms suggests that long-term survival may be linked to the ability to uptake exogenous DNA. This increase in DNA import is likely linked to an increased accumulation of biosynthetic intermediates as a source of nutrition to promote persistence during biofilm formation. Alternatively, increased import could contribute to horizontal gene transfer as a means to maintain genetic fidelity. Ongoing studies are investigating the contribution of increased DNA uptake during OM.

The enzymes involved in the early stages of the TCA cycle are absent in all sequenced strains of human-restricted *Haemophilus* suggesting that the recycling of NAD/NADH and generation of biosynthetic intermediates in metabolism are the primary outcomes of the TCA cycle. *Haemophilus* apparently uses the TCA cycle as entry points into central metabolism and amino acid metabolism (aspartate, serine, proline). In addition, an incomplete TCA cycle has been associated with increased persistence during infection.^56^ Interestingly, aconitase is exquisitely sensitive to oxidative stress.^57^ The absence of orthologs of aconitase in the TCA cycle of *Haemophilus* may provide further insight into the tolerance of *Haemophilus* to oxidative stress and killing by phagocytes.^58–62^

Pyruvate is a pivotal intermediate in the biosynthesis of amino acids. We observed increases in malic enzyme, aspartate ammonia-lyase phosphenolpyruvate carboxykinase, and a decrease in acetate coA-transferase, key enzymes involved in the metabolism in RM33 (Table 1). Interestingly, we previously observed that NTHI maintains aerobic respiration for at least 2 days in the middle ear during experimental OM through upregulation of proteins associated with the TCA cycle.^24^ Therefore, the evidence presented here suggests that NTHI also uses these amino acids for aerobic respiration during biofilm growth.

Glycogen is a highly-branched maltodextrin polymer for storage of glucose. The ability to synthesize and store intracellular glycogen has been associated with bacterial adaptation to changing and diverse environments by providing a reservoir to support bacterial survival during periods of starvation and stress.^63,64^ We observed a significant increase in the amount of the glycogen metabolic enzymes GlgA (synthase), GlgB (branch formation), and GlgX (debranching enzyme) in biofilms formed by RM33, compared with the parental strain, using both proteomic and transcriptomic approaches. Glycogen phosphorylase, GlgP, is the rate-limiting step in glycogen catabolism (Table 1). Neither the proteomic nor transcriptomic analyses indicate a significant change in GlgP production, suggesting that RM33 is producing glycogen but is also primed for catabolism as indicated by the increase in GlgX. Taken together, these data suggest that increased glycogen production may assist in the survival phenotype of RM33. Ongoing studies will determine the role of glycogen storage in biofilm formation as well as the pathogenesis and persistence of NTHI during OM.

There were significant increases in the sialic acid transporter and the acetylneuraminic acid mutarotase in RM33 biofilms (Table 1). The sialic acid transporter is known to be regulated by cAMP in NTHI^65^ and the modification of lipooligosacchride (LOS) with sialic acid provides a survival advantage in serum as well a nutrient source.^66–70^ Thus, we would predict that RM33 would also exhibit increased survival in serum as compared with the parental strain. The lipid composition of bacterial membranes is dynamic; the types and modifications of lipids can be altered in response to variations in the environment (e.g., temperature, osmotic pressure, nutrient deprivation).^71^ Phosphatidylcholine is also a metabolic precursor in bacteria and is important for virulence in a number of pathogens.^71,72^ In general, RM33 has higher levels of the phospholipids evaluated in this study (Supplementary Table 2, Supplementary Fig. 5). *Haemophilus* is one of the few bacterial species known to produce phosphorylcholine for modification of LOS, an important mediator of binding to epithelial cells.^73,74^ However, the specific contributions of each of these lipid molecules to the physiology of RM33 are not yet known.

The increase in production of the carbon starvation protein (CstA) in RM33 is consistent with the ortholog being regulated by cAMP in *E. coli*. This membrane protein transports peptides and pyruvate as a means to scavenge nutrients, enhance metabolic potential, and escape starvation,^75–77^ suggesting that CstA contributes to RM33 long-term survival.

Polyamines are metabolites that play important roles in survival. Putrescine increases biofilm formation and promotes bacterial viability, particularly in the presence of oxidative stress.^78–82^ In addition, putrescine can be catabolized through two pathways as additional nitrogen sources.^83^ Furthermore, the downstream product of putrescine degradation is succinate, which is an intermediate in the TCA cycle (Fig. 2). NTHI contains orthologs for many of the enzymes used for putrescine catabolism (Supplementary Table 3). To date, 12 enzymatic activities have been identified for the biosynthesis of the polyamine putrescine^84^ that convert either arginine or glutamate to produce ornithine.^84^ *H. influenzae* does encode the classical arginase that converts arginine to ornithine. In addition, orthologs that would convert agmatine either directly or indirectly to putrescine have not been identified in the genome of *H. influenzae*.^84^ The enzymes that convert glutamate to ornithine through two intermediate steps are present in the parental strain 86-028NP (Fig. 4).

Some bacterial species encode a putrescine-specific uptake system, *potFGHI*. However, this ABC transporter could not be identified in any of the published genome sequences for *H. influenzae*. Some strains of *H. influenzae* encode an ortholog of the *speF* ornithine decarboxylase (converts ornithine to putrescine) followed by the *potE* putrescine exporter.^85^ However, orthologs of *potE* have not been identified in the strain used in this study. The significant differences of putrescine observed in the biofilms could not be attributed to import from the environment as there was no difference in the levels of putrescine in the spent medium (Supplementary Fig. 4). Therefore, it seems likely that 86-028NP synthesizes putrescine through as yet unknown enzymatic activities.

The polyamine spermidine plays important roles for *Salmonella* survival during sepsis, in part, through mediating regulation of virulence gene expression and resistance to nitrosative stress.^86,87^ In *Streptococcus pneumoniae*, failure to import spermidine attenuates virulence due to an increase in phagocytosis by neutrophils^88,89^ and induction of autolysis.^90^ The inability to synthesize or transport spermidine also results in pleiotropic phenotypes including defects in morphological plasticity, macrophage association and early intracellular growth of *Legionella pneumophilia*.^91^ In *Vibrio cholera*, spermidine appears to be a negative regulator of biofilm formation.^92^ Spermidine also decreases susceptibility of *Pseudomonas aeruginosa* to antimicrobial agents.^93–96^ The pathoadaptation of *Shigella* results in increased resistance to oxidative stress as a consequence of increased spermidine production.^81^ Spermidine also modulates disease severity through reduction in the levels of infiltrating neutrophils, inflammatory mediators produced by stimulated macrophages and hyperplasia.^97^

There are three known pathways and thirteen enzymatic activities known to synthesize spermidine^84^ (Supplementary Table 3). A subset of sequenced *H. influenzae* strains appear to have an ortholog of the human spermidine synthase enzyme, but not 86-028NP. Spermidine, although present at 8 µM in fresh medium, is not detectible in the spent media from the biofilms, suggesting that intracellular pools of spermidine may arise from import through the PotABCD inner membrane transporter.

Our metabolomic analyses detected a significant increase in two acylcarnitines in RM33 biofilms (Supplementary Table 2). Very little is known about the biological role of acylcarnitines in bacterial physiology, particularly during biofilm growth. Acylcarnitines can be used directly as carbon, nitrogen or energy sources, or can be metabolized for entry into the TCA cycle.^98^ Carnitines are known to act as osmolytes in a number of bacterial species for osmoprotection.^98^ Carnitines are also important mediators of protection against other stressors (e.g., bile salts, cold, heat, pressure).^98^

In summary, these studies highlight the importance of sustained reprogramming of metabolism through mutational adaptation of NTHI to stressful environments such as nutrient deprivation for long-term survival during disease. Future studies will investigate the role of these metabolic processes during OM including the effects on persistence, disease progression, as well as intracellular community and biofilm lifestyles. Delineation of the metabolism of NTHI during disease will provide new avenues for the development of advanced and antibiotic sparing approaches to the prevention and treatment of this burdensome childhood infection.

## Methods

### Strains and media

For routine culturing, strains were grown on chocolate agar (Becton Dickinson, Sparks, MD). All growth was at 37 °C in 5% CO_2_. For routine liquid culture, cells were grown in BHI supplemented with 2 µg/mL of NAD and 2 µg/mL of heme (sBHI).

NTHI strain 86-028NP is a minimally passaged, fully sequenced clinical isolate that has been extensively characterized in the chinchilla model of human OM.^99–102^ NTHI strain RM33 was obtained via heme–iron restriction and isolated on day 33 of long-term culture of 86-028NP.^33^ In mixed biofilm assays 86-028NP carried the green fluorescent protein (GFP) expressing plasmid pGM1.1,^103^ while RM33 was transformed with the mCherry expressing plasmid pKM1.1.^32^

86-028NP*rpsL*∆*tnaAB* was generated using an adaptation of the recombineering method developed for *Haemophilus*.^104^ Three amplicons were generated by PCR: (i) a cassette containing a spectinomycin resistance gene and a copy of *rpsL* from *Neisseria gonorrhoeae* (*rpsL*_*Ng*_), (ii) the start codon of *tnaA* and ~1 kb of upstream DNA, (iii) the last 21nt of *tnaB* and ~1 kb of downstream DNA and pGEM-T Easy (Promega, Madison, WI; see Supplementary Table 1 for primer sequences). The *tnaA* and *tnaB* flanking amplicons had terminal 15 bp overhangs homologous to the spec^R^- *rpsL*_*Ng*_ cassette and pGEM-T Easy. In-Fusion HD (Takara Bio USA Inc., Mountain View, CA) was then used to assemble a construct in which the spec^R^-*rpsL*_*Ng*_ cassette flanked by 1 kb of DNA upstream of *tnaA* and 1 kb of DNA downstream of *tnaB* were cloned into pGEM-T Easy. This construct was linearized by restriction digest and introduced into 86-028NP*rpsL* using the MIV method.^105^ After transformation, 86-028NP*rpsL*∆*tnaAB*::spec^R^-*rpsL*_*Ng*_ clones were selected on chocolate agar supplemented with 200 µg/ml spectinomycin. The cassette was then removed by site-specific recombination to generate 86-028NP*rpsL*∆*tnaAB*.^104^ The same methodology was used to produce a construct in pGEM-T Easy in which *trpBA* was deleted and replaced by the spec^R^-*rpsL*_*Ng*_ cassette. This construct was used to transform both 86-028NP*rpsL* and 86-028NP*rpsL*∆*trpAB* to ultimately generate 86-028NP*rpsL*∆*tnaBA* and 86-028NP*rpsL*∆*trpAB*∆*tnaBA*, respectively. Each mutant was confirmed by PCR and sequencing.

### Biofilm growth on abiotic surface

Biofilm growth was assessed by growth on glass chamber slides.^32,103^ For single strain biofilms 200 µL of bacteria were added per well. For mixed biofilms, 86-028NP(pGM1.1) and RM33(pKM1.1) were combined 1:1 and 200 µL of the mix added per well. For biofilms formed by strains carrying fluorescent markers, the biofilms were mounted in ProLong Gold Antifade (Thermo Fisher Scientific). Biofilms formed by strains that did not contain a fluorescent reporter were stained with *Bac*Lite^TM^ LIVE/DEAD stain (Molecular Probes, Grand Island, NY) prior to fixation. Biofilm structure and organization were imaged with an Axiovert 200 M inverted epifluorescence microscope equipped with the Apotome attachment for improved fluorescence resolution and an Axiocam MRM CCD camera (Carl Zeiss Inc. Thornwood, NY). Three-dimensional renderings were performed to generate orthogonal views. The biofilm assay using RM33 was performed on five independent occasions and the biofilm heights in 90 random fields of view were measured. The biofilm assay using the *trp* and *tna* mutant strains was performed on three independent occasions and the biofilm height was measured from 40 random fields of view. Statistical significance was determined using a one-tailed Student’s *t* test (Graph Pad Prism, La Jolla, CA).

### Transformation efficiency experiments

To assess the transformation efficiency of the parent and RM33 isolates, a pGEM-T Easy∆sRNA121:: spec^R^-*rpsL*_*Ng*_ construct^33^ linearized by restriction digest was used to determine strain transformability of biofilms using a modified MIV method.^105^ Biofilms composed of either 86-028NP or RM33 were grown as outlined above. For each strain, all eight wells of an eight-well chamber slide were used. After 48 h of biofilm growth, the medium was removed and replaced with MIV medium containing 400 ng of linearized DNA per well. The amount of DNA used was adjusted to match the same ratio in a standard, planktonic MIV transformation. The chamber slide was incubated for 100 min at 37 °C, 5% CO_2_ after which 500 µL of sBHI was added to each well and incubated at 37 °C, 5% CO_2_ for an additional 90 min. The biofilms were then disrupted by scraping using mini-cell scrapers and all eight wells per strain were pooled. The efficiency of cell dispersion was assessed by heat fixing a sample of each strain to a microscope slide and visualizing by Gram staining. For both 86-028NP and RM33 scraping produced a uniform suspension of predominantly single cells. The pooled bacteria were grown on chocolate agar to enumerate the total viable population, as well as on chocolate agar that contained 200 µg/ml spectinomycin, to enumerate the transformed population. The transformation efficiency is reported as the percentage of transformed bacteria from the total number of viable bacteria. Four biological replicates were assayed and statistical significance was determined using a two-tailed Student’s *t* test (Graph Pad Prism, La Jolla, CA).

### Quantitation of phosphodiesterase activity

Forty-eight hour biofilms formed by strains NTHI 86-028NP and RM33 were grown in eight-well glass chamber slides and the cells harvested by scraping. The cells were centrifuged at 20,817 × *g* for 1 min at 4 °C, resuspended in 1 mL 10 mM Hepes, pH 7.3, and lysed by one passage through a high-pressure cell (20,000 psi; One Shot Model, Constant Systems Ltd., Kennesaw, GA). The protein concentration was assessed using Coomassie Protein Assay Reagent (Thermo Scientific). cAMP phosphodiesterase activity was measured using a PDELight HTS cAMP phosphodiesterase Kit (Lonza, Morristown, NJ). Thirty-nine microliters of 10 µM cAMP was mixed with 100 ng of lysate, to give a final volume of 40 µL. Samples were incubated for 30 min at room temperature then 20 μL of AMP detection reagent was added. After a further incubation for 10 min at room temperature, luminescence was quantified using a Synergy Hybrid H1 Reader (BioTek, Winooski, VT; default luminescence setting with a 0.1 s integration time). Six biological replicates were assessed in technical triplicate and statistical significance was determined using a one-tailed Student’s *t* test (Graph Pad Prism, La Jolla, CA).

### Growth and preparation of biofilms for metabolomic, transcriptomic, and proteomic analyses

Forty-eight hour biofilms of strains NTHI 86-028NP and RM33 were grown in eight-well chamber slides as described above. At 24 h post-inoculation, supernatants were collected from each well and pooled for each strain. The 24 h supernatants were centrifuged at 20,817 × *g* for 5 min to remove any bacterial cells and the supernatants were transferred into new microcentrifuge tubes and stored at −80 °C. Forty-eight hours postinoculation, the biofilm supernatants were collected from each well and pooled for each strain. Supernatants were centrifuged, transferred to new microcentrifuge tubes, and frozen at −80 °C. Supernatants were collected to control for the secretion and/or depletion of metabolites into and from the medium. Forty-eight hour biofilms were then washed once with 200 μL of 50 mM ammonium bicarbonate (pH 8.0) before being resuspended in 200 μL 50 mM ammonium bicarbonate. Mini-cell scrapers were used to ensure collection of entire biofilm from each well. Biofilms from all eight wells were pooled for each strain and each sample was subjected to two passages through a high-pressure cell (20,000 psi; One Shot Model, Constant Systems, Ltd., Kennesaw, GA). Protein concentrations for each sample were then determined using the Pierce Coomassie Protein Assay Kit (Thermo Fisher Scientific) and samples were stored at −80 °C prior to analyses. Three biological replicates were processed for each biofilm.

### Proteomics

#### Label-free Quantitative Analysis of NTHI biofilm proteins

Quantitative liquid chromatography-tandem mass spectrometry (LC-MS/MS) was performed in singlicate on 0.3 µg of protein digest per sample, and the pool was analyzed four times with 0.3 μg injections spaced evenly across the run queue using the acquisition method immediately described below. The method used liquid chromatography on a nanoACQUITY UPLC system (Waters Corp, Milford, MA) coupled to a Q-Exactive Plus high-resolution accurate mass tandem mass spectrometer (Thermo Fisher Scientific) with nanoelectrospray ionization. Mobile phase A and B consisted of 0.1% formic acid (v/v) in water and acetonitrile, respectively. Peptides were first trapped at 5 µl/min, 99.9%A, on a 5 µm Symmetry C18 180 µm × 20 mm trapping column (Waters Corp). Analytical separations were performed on a 1.8 µm Acquity HSS T3 C18 75 µm × 250 mm column (Waters Corp) using a linear gradient of 5–40% B over 90 min, at a flow rate of 0.4 µl/min and column temperature of 55 °C. Data collection on the Q-Exactive Plus mass spectrometer were performed in data-dependent acquisition mode of acquisition with Rs = 70,000 (@ m/z 200) full MS scan from m/z 375 to 1600 with a target AGC value of 1e6 ions followed by 10 MS/MS scans at Rs = 17,500 (@ m/z 200) at a target AGC value of 5e4 ions. A 20 s dynamic exclusion was employed.

#### Analysis of proteins

Data were imported into Rosetta Elucidator v3.3 (Rosetta Biosoftware, Inc, Seattle, WA), and all LC-MS runs were aligned based on the accurate mass and retention time of detected ions (“features”) using PeakTeller algorithm (Elucidator). The relative peptide abundance was calculated based on area-under-the-curve (AUC) of aligned features across all runs. The MS/MS data were searched against a custom database containing NCBI *H. influenzae* sequences; the database also contained a reversed-sequence “decoy” database for false positive rate determination as well as several proteins which were surrogate standards or common contaminants. Included in the database searches were variable modifications on M (oxidation) and N/Q (deamidation), and fixed modification on C (carbamidomethyl). After individual peptide scoring using PeptideProphet algorithm (Elucidator), the data were annotated at a 1% peptide false discovery rate. This analysis yielded identifications for 6213 peptides and 1207 proteins across all samples. Seven hundred sixty-nine proteins were identified and quantified with 2 + peptides to match. For quantitative processing, filters were applied to ensure peptides had good chromatographic peak shape and good MS spectral integrity. For this dataset, no peptides were removed and the final quantitative dataset contained 6213 peptides and 1207 proteins. The protein-level summarization (e.g., protein-level expression data) is presented in Table 1. Principal component analyses of the individual and pooled samples demonstrate segregation of the samples within each condition as well as technical fidelity of the pooled samples (Supplementary Fig. 1). The raw and processed mass spectrometry proteomics data have been made available via the MassIVE data repository at ftp://massive.ucsd.edu/MSV000084166/.

### Metabolomics

Biofilm lysates and 48 h spent supernatants were prepared and analyzed for metabolites.^24^ Biofilm lysates and culture supernatants were prepared using the Absolute*IDQ* p180 kit (Biocrates Innsbruck, Austria) following the manufacturer protocol. Ten microliters of a solution containing stable-isotope labeled internal standards and 15 µl of each biofilm lysate were added to a 96-well extraction plate and the plate dried under a gentle stream of nitrogen. An additional 15 µl of each biofilm lysate and culture supernatant were then added to the wells and the drying process repeated. The samples were derivatized with phenyl isothiocyanate then eluted with unbuffered 5 mM ammonium acetate in methanol. Samples were diluted 4:1 v/v (5× dilution) with 40% methanol in water for ultra performance liquid chromatography (UPLC) analysis or 19:1 v/v (20× dilution) with running solvent (a proprietary mixture provided by Biocrates) for flow injection analysis. A QC pool based on equal volumes of all samples was created. The pooled sample was prepared exactly as the experimental samples and injected once before, once during, and once after the experimental samples in order to measure the performance of the assay during sample analysis.

#### Quantitative analysis of metabolites from biofilm lysates and culture supernatants

Separation of amino acids and biogenic amines was performed using an ACQUITY UPLC System (Waters Corporation) using an ACQUITY 2.1 mm × 50 mm 1.7 µm BEH C18 column fitted with an ACQUITY BEH C18 1.7 µm VanGuard guard column, and quantified by calibration curve using a linear regression with 1/x fit. Acylcarnitines, sphingolipids, and glycerophospholipids, were analyzed by flow injection analysis tandem mass spectrometry (FIA-MS/MS), quantified by ratio to a stable-isotope labeled internal standard in the same analyte class. Thus, FIA analytes are reported as semiquantitative values except where a stable-isotope labeled internal standard of the exact analyte was used. Samples for both UPLC and flow injection analysis were introduced directly into a Xevo TQ-S mass spectrometer (Waters Corporation) using positive electrospray ionization operating in the Multiple Reaction Monitoring (MRM) mode. MRM transitions (compound-specific precursor to product ion transitions) for each analyte and internal standard were collected over the appropriate retention time using tune files and acquisition methods provided in the AbsoluteIDQ p180 kit from Biocrates. The UPLC data were imported into TargetLynx (Waters Corporation) for peak integration, calibration, and concentration calculations. The UPLC data from TargetLynx and flow injection data were analyzed using Biocrates’ Met*IDQ* software. The data for the p180 Kit are reported in Supplementary Table 2. Each analyte was normalized for the total protein in the sample, concentrations shown Supplementary Table 2. Principal component analyses of the samples demonstrates the segregation of the two strains as well as the technical fidelity in the pooled samples (Supplementary Fig. 2).

### RNA isolation and purification

Total RNA was isolated from 48 h biofilms using a modified hot phenol method.^106^ All centrifugation steps were at 5700 × *g* and at 4 °C, unless otherwise stated. Biofilms were suspended in sBHI and scraped from chambers using mini-cell scrapers and pooled. The resuspended biofilms were then mixed with one volume of acid phenol containing 0.1% SDS. The tubes were heated at 90 °C until the phases merged and then chilled on ice until the phases separated. The tubes were centrifuged for 20 min, the aqueous layer removed and mixed with an equal volume of acid phenol:chloroform until an emulsion formed. The tubes were then centrifuged for 15 min. The aqueous layer was removed and mixed with an equal volume of chloroform until an emulsion formed and then centrifuged for 15 min. The aqueous phase was removed, mixed with 1/10 volume of 3 M sodium acetate and one volume of isopropanol then incubated overnight at −20 °C. RNA was pelleted by centrifugation for 15 min followed by resuspension in 1 mL 70% ethanol and then centrifuged at 20,817 × *g* for 1 min at 4 °C. The pellet was washed in 500 µL 70% ethanol and centrifuged at 20,817 × *g* for 1 min at 4 °C prior to being dried. To remove DNA the RNA was incubated for 2 h at 37 °C with 15 units of DNaseI (New England Biolabs, Ipswich, MA) and 20 units of SUPERase In RNase inhibitor (ThermoFisher Scientific). After digestion, the RNA was purified using an RNeasy Mini Kit (QIAGEN). Purified RNA was quantified using a Nanodrop ND-1000 Spectrophotometer (ThermoFisher Scientific) and quality assessed using an Agilent 2100 Bioanalyzer (Agilent, Santa Clara, CA) with an Agilent RNA 6000 Nano Kit (Agilent).

### Illumina HiSeq 2500 sequencing

Two hundred nanograms of the RNA was fragmented to 200 bp by Covaris (Covaris Inc, Woburn MA) and cDNA synthesized. The double-stranded cDNA was end repaired, adenylated, ligated to standard Illumina adapters then amplified by PCR. The RNA-Seq library was validated using the Qubit fluorometer for mass and concentration and analyzed using the Agilent 2100 Bioanalyzer for concentration and insert size to assure library loading concentration and integrity prior to being sequenced using an Illumina HiSeq 2500 sequencer with the HiSeq Rapid SBS kit v2 (Illumina). 1 × 100 bp single-end sequencing was conducted across one flow cell lane with a multiplex of eight samples in one lane. Raw data has been uploaded to the National Center for Biotechnology Information (NCBI) Gene Expression Omnibus (GEO) (GSE131505). Image analysis and base calling were performed with Sequence Analysis Viewer tool. Demultiplexing was performed with bcl2fastq (Illumina) prior to data analysis. Four replicates for each strain were sequenced, with an average read count of 43,936,303 for 86-028NP and 48,414,002 for RM33. Each sample was aligned to the 86-028NP reference genome (NCBI; GCA_000012185.1) using the BWA-MEM aligner (version 0.7.15; http://bio-bwa.sourceforge.net/). Feature coverage counts were calculated using HTSeq^107^ and differentially expressed features were calculated using DESeq2.^108^

### Indole assay

Extracellular indole concentration was measured using a modified version of a published.^109^ Bacterial strains were grown to mid-logarithmic phase (OD_490_) in sBHI medium at 37 °C with 5% CO_2_. Cultures were diluted 1:2500 and 200 μL placed in each well of three eight-well chamber slides per strain. Following incubation at 37 °C with 5% CO_2_ for 24 or 48 h, biofilm supernatants from each well were collected and pooled for each strain. For 48-h biofilms, a media change was performed at 24 h. To determine indole concentration, 2.5 mL biofilm supernatant was mixed with 1 mL of HCl:n-Butanol (50 mL HCl + 150 mL n-Butanol) and allowed to become biphasic. One hundred and fifty microliters of the organic phase (containing extracted indole) was removed and placed in quadruplicate in a clear, flat bottom 96-well plate. Sixty microliters Kovac’s Reagent (Sigma Aldrich) was then added to each well and incubated for 2 min. A replicate without Kovac’s reagent was included as a control for each sample. Absorbance at 570 nm was immediately read using a spectrophotometer. To quantify indole concentration, a standard curve was created as follows. One milligram of indole (Sigma Aldrich) was dissolved in 1 mL HCl:n-Butanol in glass tubes and serially diluted to generate a standard curve ranging from 1 to 0 mg indole. Two and a half milliliters of freshly prepared sBHI was added to each tube and the mixture was allowed to become biphasic. One hundred and fifty microliters of the organic phase was removed and placed in quadruplicate in the same microtiter plate. Sixty microliters Kovac’s reagent was added to each well and incubated for 2 min. A replicate without Kovac’s reagent was included as a control for each standard concentration. Absorbance at 570 nm was immediately read using a spectrophotometer. Indole concentration in experimental samples was determined using a linear trend line based on the standard curve. Experiments were performed in technical triplicate on three independent occasions and statistical significance was determined using a two-tailed unpaired Student’s *t* test (Graph Pad Prism, La Jolla, CA).

### Reporting summary

Further information on research design is available in the Nature Research Reporting Summary linked to this article.

## Supplementary information

Supplemental material

Reporting Summary Checklist

## Data Availability

The raw and processed mass spectrometry proteomics data have been made available via the MassIVE data repository at ftp://massive.ucsd.edu/MSV000084166/. Raw data for the transcriptomics have been uploaded to the NCBI GEO (GSE131505).
