# Supplementary material for: Reprioritization of biofilm metabolism is associated with nutrient adaptation and long-term survival of Haemophilus influenzae
Source: NPJ Biofilms Microbiomes. 2019 Nov 5;5:33. doi: 10.1038/s41522-019-0105-6 (PMC6831627; doi:10.1038/s41522-019-0105-6)
Supplement: Supplementary file 1 — Supplemental material [file 41522_2019_105_MOESM1_ESM.pdf]

Proteomic analyses of RM33 and Parental Biofilms

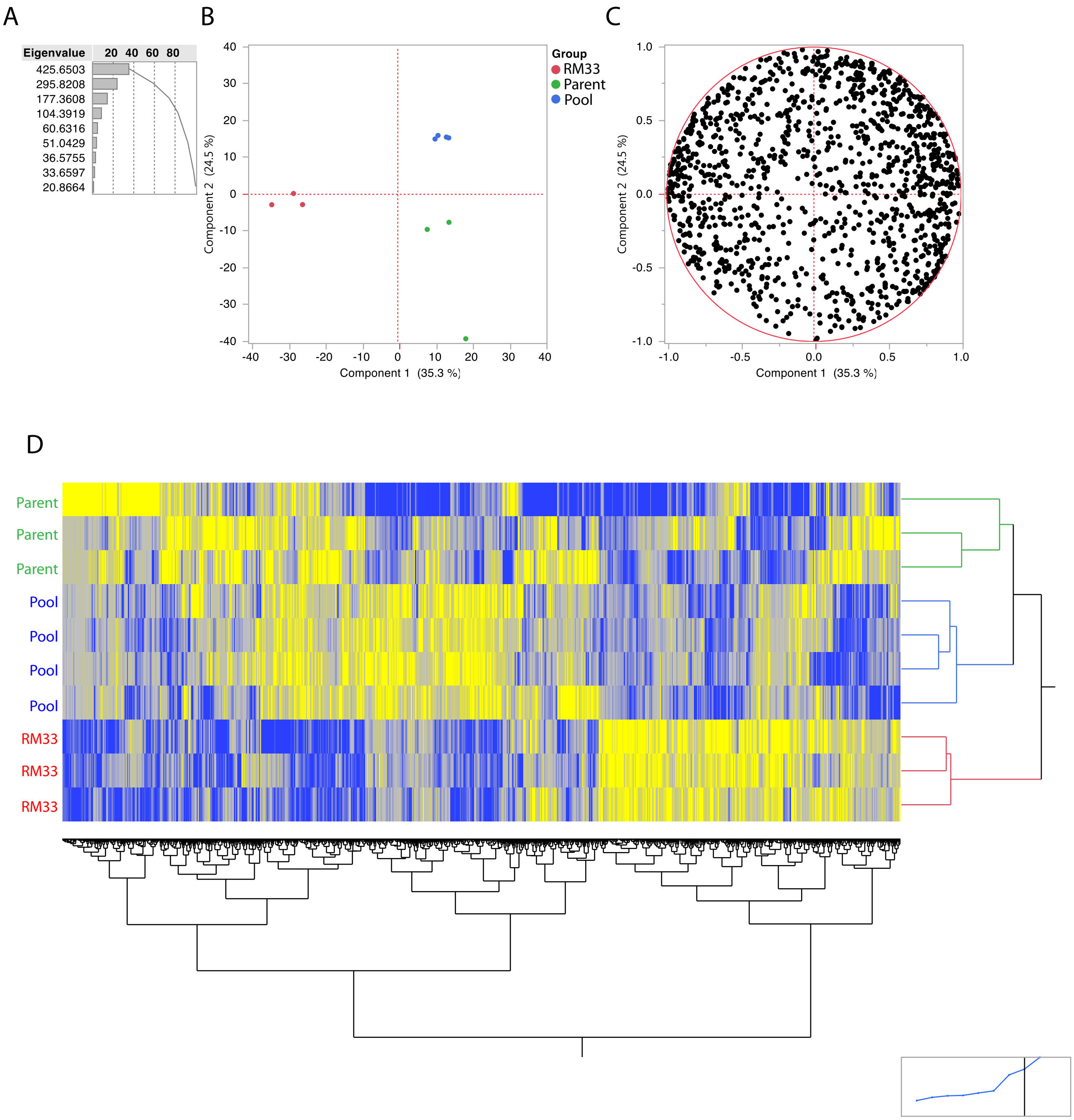

Supplemental Figure 1. **Proteomic analyses of RM33 and parental biofilms**. (A, B, C) Principal component analyses of the proteomes from the RM33 (red) and parental 86-028NP (green) samples taken from three independent biological replicates. The pool (blue) is a quality control where equivalent volumes of each sample (5 µl) were combined and analyzed in three parallel trials for quality control purposes and demonstrate high technical fidelity. (D) Dendrogram of the proteins that are increased (yellow) or decreased (blue) in the RM33 biofilms as compared with the parental strain, 86-028NP. The "pool" analyses also demonstrate strong similarity as an indicator of technical reproducibility.

Metabolomic analyses of RM33 and Parental Biofilms

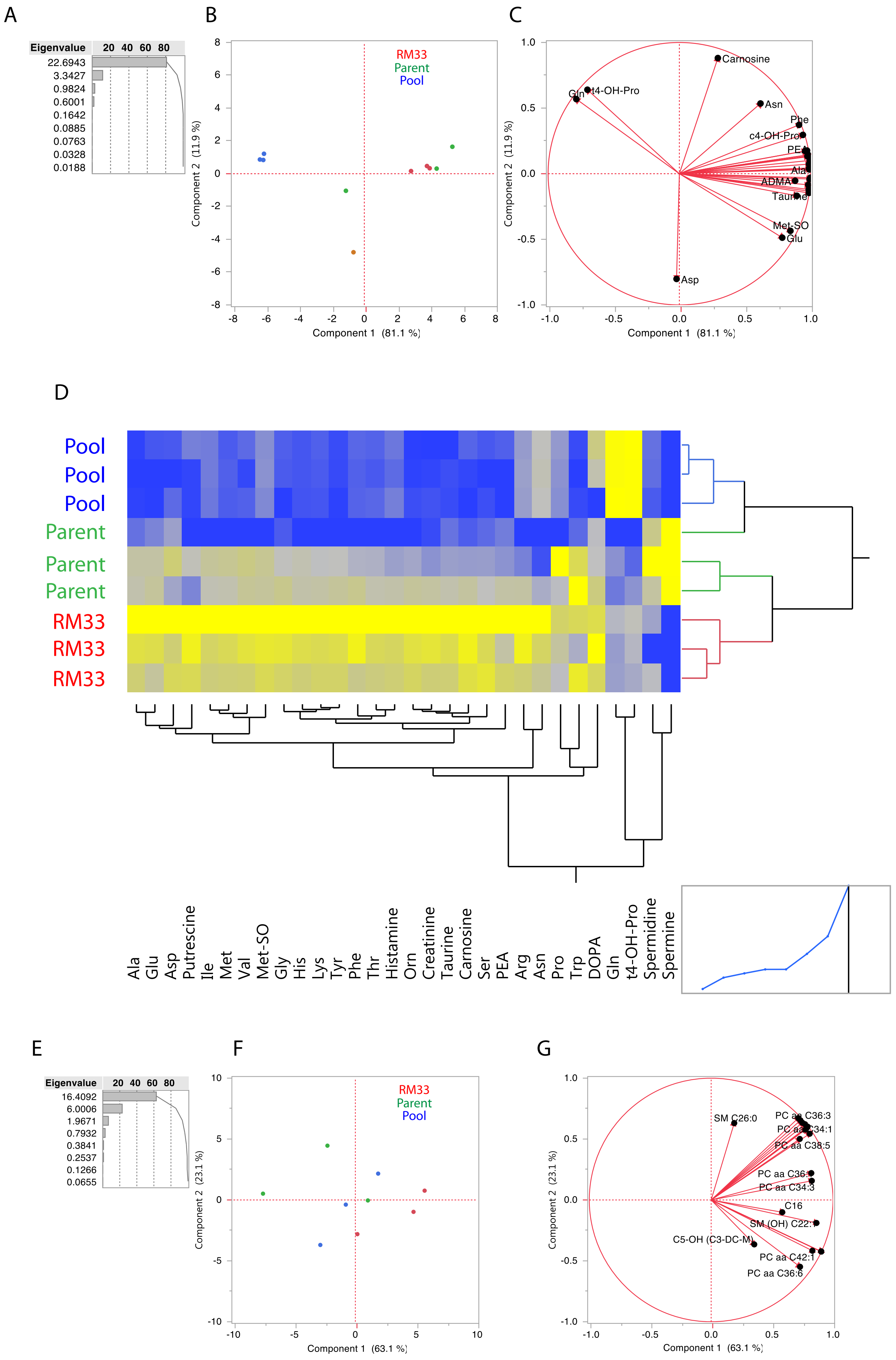

Supplemental Figure 2. **Metabolomic analyses of RM33 and parental biofilms**. (A, B, C) Principal component analyses of the metabolomes (UPLC) from the RM33 (red) and parental 86-028NP (green) samples taken from three independent biological replicates. The pool (blue) is a quality control where equivalent volumes of each sample (5 µl) were combined and analyzed in three parallel trials for quality control purposes and demonstrate high technical fidelity. (D) Dendrogram of the proteins that are increased (yellow) or decreased (blue) in the RM33 biofilms as compared with the parental strain, 86-028NP. The "pool" samples also demonstrate strong similarity as an indicator of technical reproducibility. (E, F, G) Principal component analyses of the metabolomes (FIA) from the RM33 (red) and parental 86-028NP.

Amino Acids Spent Culture/Fresh Media

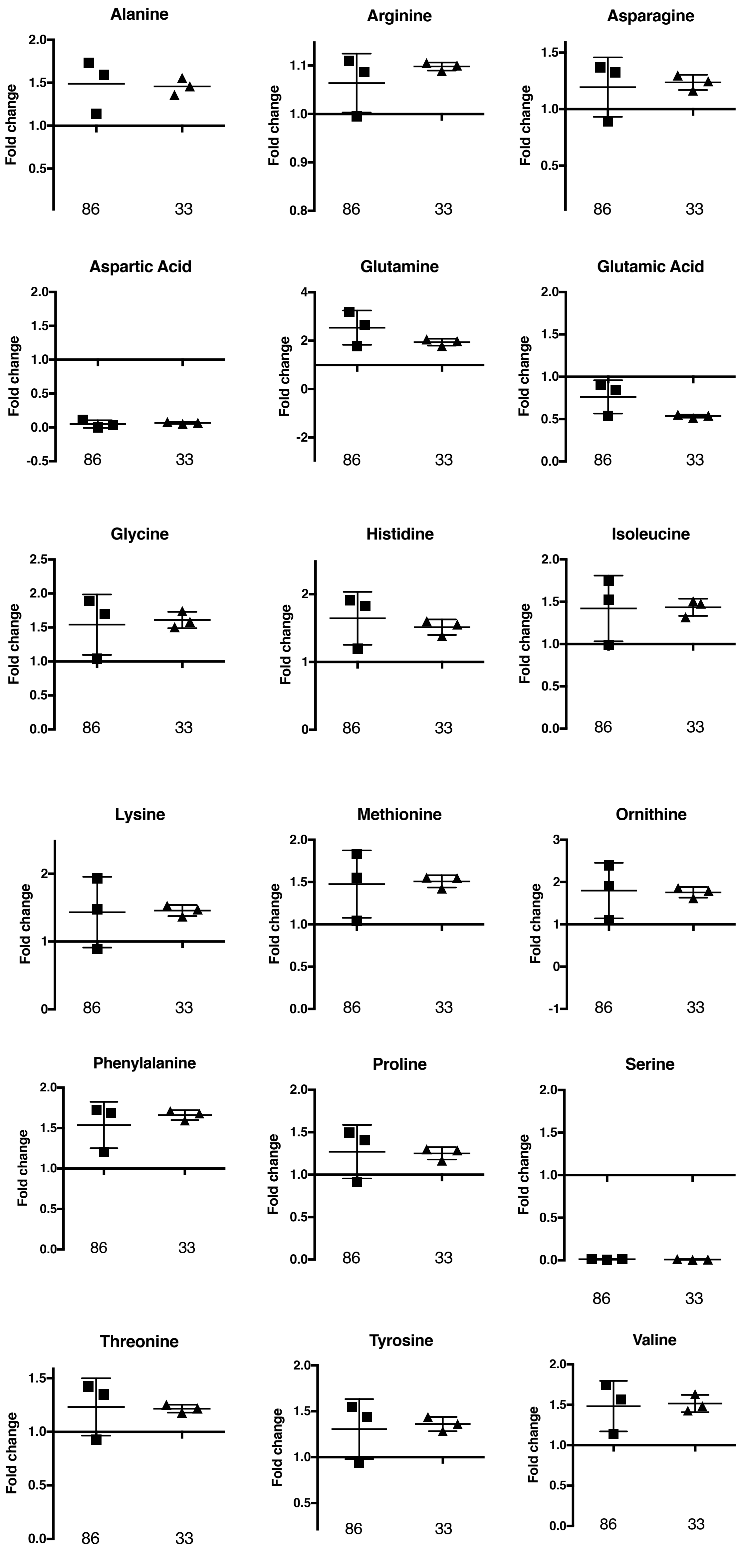

Supplemental Figure 3. **Amino acids present in the spent culture supernatant**. The extracellular concentration for each of the amino acids is reported as the ratio of fresh media over spent media. Statistical significance was determined using a two-tailed unpaired student's T-test.

Biogenic Amines Spent Culture/Fresh Media

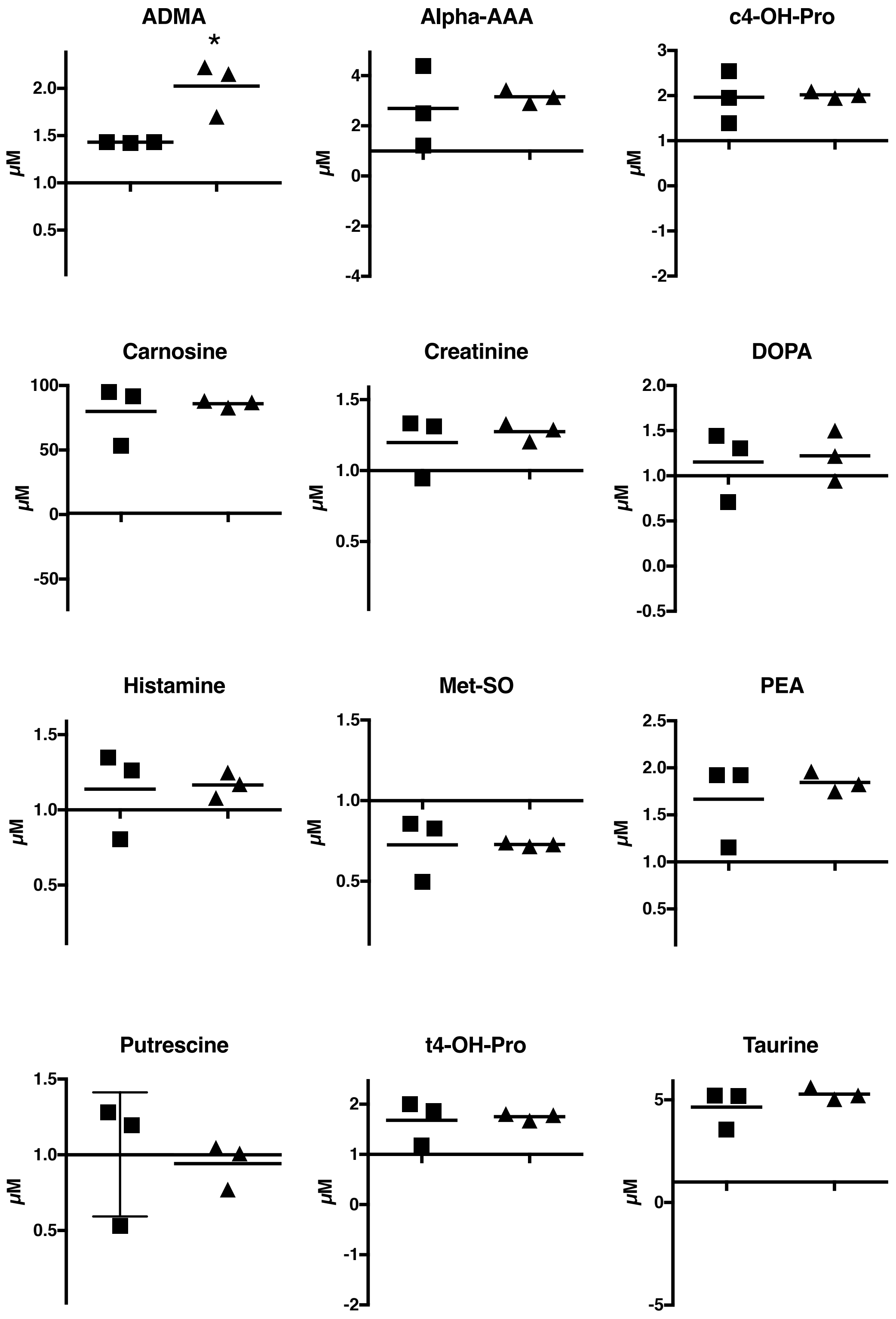

Supplemental Figure 4. **Biogenic amines present in the spent culture supernatant**. The extracellular concentration for each of the biogenic amines is reported as the ratio of fresh media over spent media. Statistical significance was determined using a two-tailed unpaired student's T-test.

Acylcarnitines and PC
Spent Culture/Fresh Media

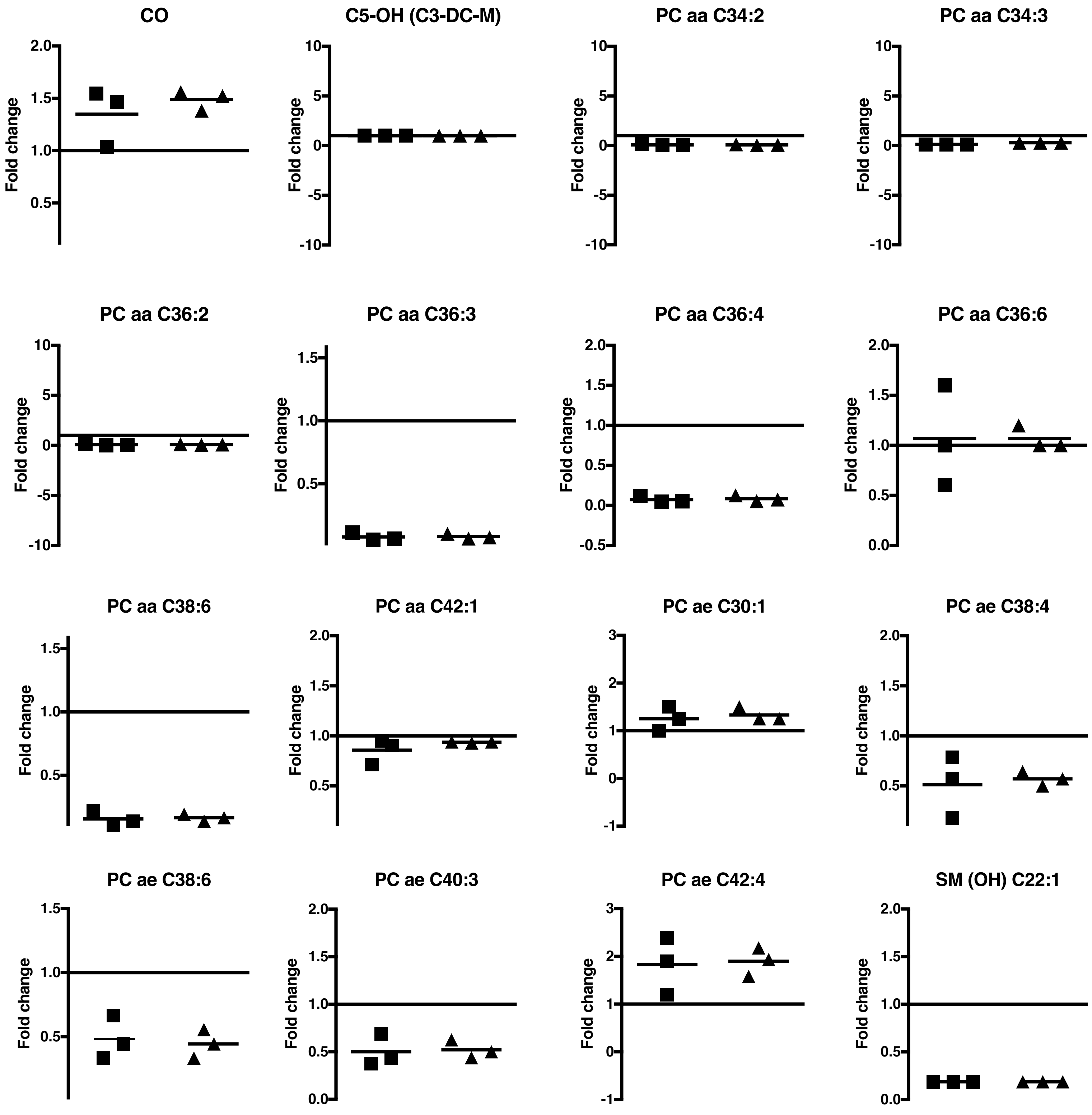

Supplemental Figure 5. **Acylcarnatines and glycerophopholipids in the spent culture supernatant.** The extracellular concentration for each of the acylcarnatines and glycerophospholipid is reported as the ratio of fresh media over spent media. Statistical significance was determined using a two-tailed unpaired student's T-test.

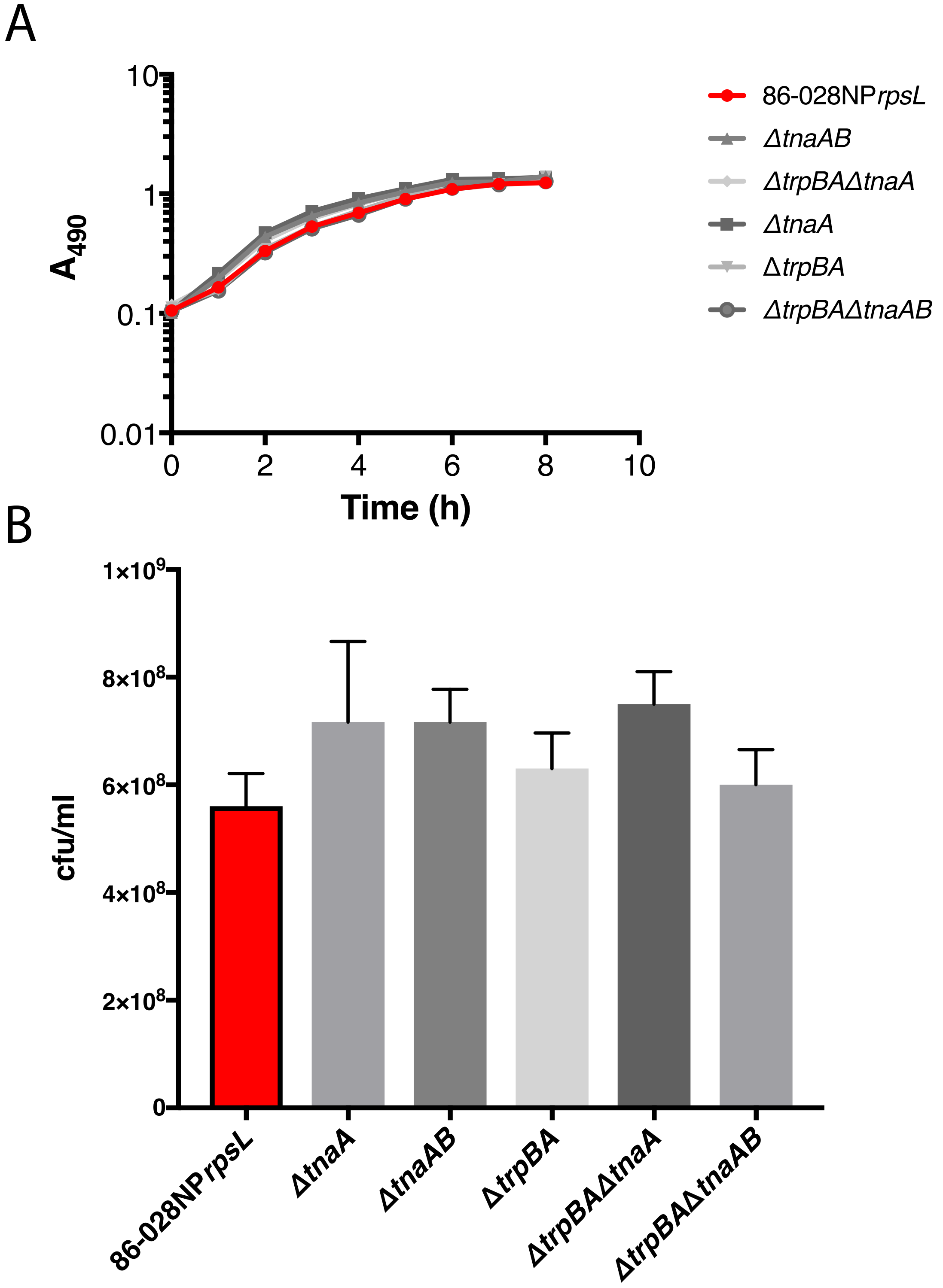

Supplemental Figure 6. **Growth of strains defective in tryptophan biosynthesis.** (A) The growth rate of each individual mutant strain was determined by growth at 37C in sBHI with measurements of the absorbance at 490 nm every hour. Representative data from 3 independent experiments. (B) Samples were taken at the three hour time point to determine the viability of each strain. (n=3 independent experiments). Statistical significance was determined using a two-tailed unpaired T-test, no significant difference was observed.

Supplemental Table 1 **Primers used in this study**

Primer	Sequence	Function
AH0838	CCGGCCGCCATGGCG**ATTGCTTACTTAGCAGGAAT**	In-Fusion primers to delete *tnaAB*
AH0839	ACGGATCCCCGGAAT**CATAGCAAAATCCCCAAAAA**	
AH0840	CAGCTCCAGCCTACA**TTACCTTTCTTTAAGTTCTAA**	
AH0841	CGCGAATTCACTAGTT**TCGTCCAACTCCGCATGGT**	
AH0842	CCGGCCGCCATGGCG**CTTTGATACGTTCGACTAAT**	In-Fusion primers to delete *trpBA*
AH0843	ACGGATCCCCGGAAT**CATATTTTTTCCTATTATTT**	
AH0844	CAGCTCCAGCCTACA**ATGAAAGCTGCAACAAAATA**	
AH0845	CGCGAATTCACTAGT**GGTGTAAAATAATGCTGATT**	
AH0846	CGCCATGGCGGCCGGGAGCATG	In-Fusion primers to amplify pGEM-T Easy
AH0847	ACTAGTGAATTCGCGGCCGCCTGCA	
AH0848	TGTAGGCTGGAGCTGCTTCG	In-Fusion primers to amplify specR-$rpsL_{Ng}$
AH0849	ATTCCGGGGATCCGTCGACC	

Amino Acids
(µmol/g protein)

	86-028NP	RM33	*p* value
**Alanine**	**4714 ± 610**	**7785 ± 772**	**0.0355**
**Arginine**	**5898 ± 1190**	**11367 ± 952**	**0.0230**
**Asparagine**	**628 ± 85**	**1242 ± 137**	**0.0188**
Aspartic acid	947 ± 132	1390 ± 127	0.0730
Glutamine	430 ± 69	651 ± 53	0.0642
Glutamic acid	1572 ± 126	2036 ± 181	0.1036
**Glycine**	**3850 ± 631**	**6330 ± 492**	**0.0363**
**Histidine**	**872 ± 124**	**1395 ± 127**	**0.0417**
Isoleucine	2276 ± 395	3537 ± 348	0.0749
**Lysine**	**8215 ± 1331**	**14582 ± 1389**	**0.0297**
Methionine	959 ± 164	1496 ± 139	0.0667
**Ornithine**	**399 ± 72**	**766 ± 70**	**0.0219**
Phenylalanine	2357 ± 432	4040 ± 464	0.0566
Proline	651 ± 162	673 ± 53	0.9020
**Serine**	**506 ± 39**	**899 ± 54**	**0.0042**
**Threonine**	**1130 ± 150**	**1935 ± 207**	**0.0345**
Tryptophan	73 ± 17	73 ± 11	0.9874
**Tyrosine**	**1190 ± 193**	**2018 ± 178**	**0.0343**
Valine	2667 ± 482	3898 ± 293	0.0944

Biogenic Amines
(µmol/g protein)

	86-028NP	RM33	*p* value
Ac-Orn	ND	ND	
ADMA	ND	ND	
alpha-AAA	ND	ND	

c4-OH-Pro	ND	ND	
**Carnosine**	**22 ± 4**	**45 ± 3**	**0.0077**
**Creatinine**	**72 ± 14**	**158 ± 21**	**0.0259**
DOPA	3 ± 1	7 ± 1	0.0514
Dopamine	ND	ND	
**Histamine**	**10 ± 2**	**19 ± 2**	**0.0345**
Kynurenine	ND	ND	
Met-SO	177 ± 42	294 ± 17	0.0603
Nitro-Tyr	ND	ND	
PEA	ND	ND	
**Putrescine**	**70 ± 12**	**176 ± 15**	**0.0051**
Sarcosine	ND	ND	
Serotonin	ND	ND	
**Spermidine**	**255 ± 55**	**135 ± 14**	**0.0198**
Spermine	16 ± 0	ND	
**t4-OH-Pro**	**32 ± 6**	**66 ± 6**	**0.0184**
**Taurine**	**91 ± 13**	**195 ± 27**	**0.0250**

Acylcarnitines and Phosphatidylcholine

(μmol/g protein)

	86-028NP	RM33	*p* value
**C16**	**0.54 ± 0.14**	**1.2 ± 0.08**	**0.0154**
**C5-OH (C3-DC-M)**	**0.51± 0.12**	**1.00 ± 0.10**	**0.0376**
**PC aa C32:0**	**0.09 ± 0.02**	**0.18 ± 0.02**	**0.0377**
**PC aa C32:1**	**0.05 ± 0.01**	**0.11 ± 0.01**	**0.0378**
**PC aa C32:3**	**0.05 ± 0.01**	**0.11 ± 0.01**	**0.0378**
PC aa C34:1	0.47 ± 0.17	0.73 ± 0.32	0.5159
PC aa C34:2	1.02 ± 0.37	1.62 ± 0.68	0.4831
PC aa C34:3	0.04 ± 0.01	0.07 ± 0.02	0.2258

**PC aa C34:4**	**0.05 ± 0.01**	**0.09 ± 0.01**	**0.0389**
**PC aa C36:0**	**3.4 ± 0.82**	**6.64 ± 0.66**	**0.0377**
PC aa C36:1	0.10 ± 0.02	0.16 ± 0.02	0.1228
PC aa C36:2	0.6 ± 0.22	0.93 ± 0.37	0.4873
PC aa C36:3	0.32 ± 0.11	0.50 ± 0.21	0.4864
PC aa C36:4	0.54 ± 0.19	0.83 ± 0.36	0.5142
**PC aa C36:5**	**0.05 ± 0.01**	**0.09 ± 0.009**	**0.0389**
**PC aa C36:6**	**0.01 ± 0.003**	**0.03 ± 0.004**	**0.0247**
**PC aa C38:0**	**0.26 ± 0.06**	**0.51 ± 0.05**	**0.0376**
**PC aa C38:3**	**0.09 ± 0.02**	**0.17 ± 0.02**	**0.0380**
PC aa C38:4	0.30 ± 0.13	0.46 ± 0.22	0.5524
PC aa C38:5	0.11 ± 0.05	0.18 ± 0.06	0.4394
PC aa C38:6	0.09 ± 0.03	0.16 ± 0.06	0.3586
**PC aa C40:1**	**2.76 ± 0.67**	**5.39 ± 0.54**	**0.0377**
**PC aa C42:1**	**0.92 ± 0.34**	**2.19 ± 0.28**	**0.0445**
**SM (OH) C22:1**	**0.11 ± 0.03**	**0.21 ± 0.02**	**0.0380**
SM C24:1	0.38 ± 0.18	0.52 ± 0.24	0.6562
SM C26:0	0.03 ± 0.01	0.04 ± 0.01	0.8758

Supplemental Table 2. Summary of the quantification of metabolites in biofilms produced by the parent (86-028NP) or RM33. Red rows indicate statistical significance determined by two-tailed student's t test. Gray rows indicate metabolites that were below the limit of detection.

Enzyme Activity	Protein Fold	Exemplar Protein ID	HI strains	86-028NP	Query Cover	Identity
**Arginine decarboxylase**	AAT with N-terminal REC domain, acid-inducible	*Escherichia coli* AdiA (NP_418541)	5	No	88%	32%
	AAT without N-terminal REC domain	*Bacillus subtilis*, SpeA (NP_389346)	5	No	88%	23%
	AR ancestral	*Chloroflexus auranticus* (YP_001634722)	0	No	0	0
	AR with four-helical bundle insertion	*Arabidopsis thaliana* ADC (AAB72179)	0	No	0	0
		Vibrio vulnificus ADC (Q87JS8.1)	17	Yes (*lysA*, NTHI0884)	47%	25%
	AR ODC-like	*Paramecium bursaria* cholerella virus-1 (NP_048554)	0	No	0	0
	Pyruvolyl-dependent biosynthetic	*Methanococcus jannaschi* (Q57764)	0	No	0	0
	Pyruvoyl-dependent acid resistance	*Chlamydophila pneumonia* (WP_010883665)	0	0	0	0
	AdoMetDC paralog	*Sulfolobus solfataricus* (Q9UWU1)	0	0	0	0
**Ornithine decarboxylase**	AAT biosynthetic	*E. coli* SpeC (NP_417440)	6	No	97%	66%
	AAT acid-inducible	*Lactobacillus sp* SpeF (CAX67648)	5	No	88%	45%
	AR ornithine-specific	*Homo sapiens* (AAA59967)	0	0	0	0
	AR lysine-specific	*V. vulnificus* (AXX62769.1)		Yes (*lysA*, NTHI0884)	88%	27%

**Lysine decarboxylase**	AAT constitutive	*E. coli* LdcC (NP_414728)	17	Yes (6%, NTHI1577)	11%	35%
	AAT acid-inducible	*E. coli* CadA (AAA97031)	5	No	82%	35%
	AAT DABA DC paralogue	*Streptomyces coelicolor* (NP_627012)	17	Yes (*ddc*, NTHI1119)	94%	36%
	AR lysine-ornithine	*V. vulnificus* (2PLJ_A)	16	Yes (lysA, NTHI0884)	92%	26%

Enzyme Activity	Protein Fold	Exemplar Protein ID	HI strains	86-028NP	Query Cover	Identity
**AdoMetDC**	Pyruvoyl-dependent	*E. coli* SpeD (NP_414662)	0	0	0	0
	Class 1A	*Thermotoga maritima* SpeD (WP_004081137)	0	0	0	0
	Class 1B	*H. sapiens* (P17707)	0	0	0	0
**Aminopropyl transferase**	Spermidine synthase	*H. sapiens* (NP_003123)	0	0	0	0
		T. maritima (AKE28440)	0	0	0	0
		Caenorhabditis elegans (CAC37332)	0	0	0	0
		Trypanosoma cruzi (4YV0_D)	0	0	0	0
		Plasmodium falciparum (CAB71155)	0	0	0	0

		E. coli (BAB96695)	0	0	0	0
	Agmatine/triamine aminopropyltransferase	*T. thermophilus* SpeE (3ANX_B)	17	Yes (*ackA*, NTHI1375)	41%	26%
	Spermine Synthase	*H. sapiens* (NP_415220)	6	No	97%	66%
	Tspm synthase	*A. thaliana* Act5 (AED92722)	0	0	0	0
**Carboxytriamine dehydrogenase**	CANDH	*V. cholera* (NP_231262)	0	0	0	0
	CASDH	*Campylobacter jejuni* (AJP34684)	0	0	0	0
		A. tumerfaciens C58(atu4170)	0	0	0	0
**Carboxytriamine decarboxylase**	CANDC	*V. cholera* (NP_231262)	0	0	0	0
	CASDC	*C. jenjuni* (A8FNH9)	0	0	0	0
		A tumerfaciens C58(atu4169)	0	0	0	0
**Homospermidine synthase**	HSS	*Legionella pneumophilia* (ADG25880)	0	0	0	0
		Blastochloris viridis (AAB63957.1)	0	0	0	0
	DHS	*H. sapiens*				
		Senecia vulgaris (CAB66389)	0	0	0	0

Supplemental Table 3. List of known proteins involved in polyamine biosynthesis. Adapted from information provided in reference 84.
